# CO and NO_2_ dynamics in a Brazilian tropical transition zone: the role of land use and road infrastructure

**DOI:** 10.1007/s11356-026-38001-6

**Published:** 2026-07-13

**Authors:** Alline Gomes Lamenha e Silva, Mônica Regina Garcez, Lélio Antônio Teixeira Brito

**Affiliations:** 1 Environment Department, Federal Institute of Education Science and Technology of Alagoas: Instituto Federal de Educacao Ciencia e Tecnologia de Alagoas, Eng. Joaquim Gonçalves Highway - Dom Constantino, Penedo, Alagoas 57200-000 Brazil; 2 Pavement Laboratory (LAPAV), Federal University of Rio Grande Do Sul: Universidade Federal Do Rio Grande Do Sul, Bento Gonçalves Avenue, 9500, Building 43816 - Agronomia, Porto Alegre, Rio Grande do Sul 91501-970 Brazil

**Keywords:** Air pollutants, Land use and land cover, Vehicular emissions, Prophet forecasting

## Abstract

Land-use dynamics and road infrastructure are key anthropogenic drivers of air pollution in tropical landscapes undergoing rapid transformation. This study investigates how land cover and proximity to major roads influence carbon monoxide (CO) and nitrogen dioxide (NO₂) concentrations in a transitional region between the Amazon and Cerrado biomes, in Brazil. Sentinel-5P (TROPOMI) satellite data from December 2018 to August 2024 were used to analyze spatial patterns, test statistical differences across land-use/cover (LULC) classes and highway buffer zones, and forecast pollutant trends for 2024–2025 using the Prophet model. Results demonstrate that LULC influences pollutant variability: CO concentrations respond most strongly to biomass-burning episodes, whereas emissions from urban areas and vehicle traffic along transportation corridors primarily influence NO₂. Forecasts predict rising pollutant burdens across all subsets, with CO growth rates ranging from 2.7% y⁻^1^ in non-vegetated areas to 4.0% y⁻^1^ in herbaceous and shrubby vegetation (model *R*^2^ = 0.90–0.94) and NO₂ growth rates from 0.3% y⁻^1^ in herbaceous and shrubby vegetation to 1.0% y⁻^1^ in farming and highway buffer zone (model *R*^2^ = 0.91–0.93). The findings contribute to air quality management by linking land-use strategies and transport planning to pollutant behavior, with implications for environmental policy in tropical and subtropical regions.

**Graphical abstract:**

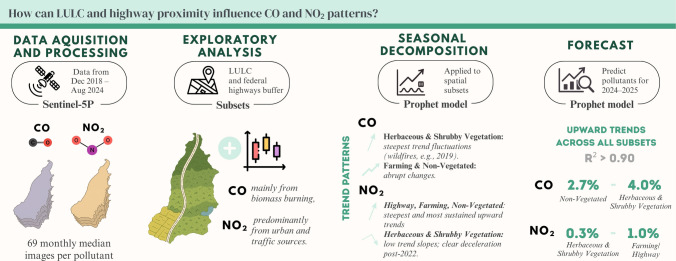

## Introduction

Human activity has become the main force reshaping Earth’s ecosystems, changing land cover and atmospheric conditions at various scales (Small and Sousa 2016; Thomas et al. 2020). These transformations are not only disrupting ecological balances but also increasing human exposure to environmental hazards.

Among them, air pollution stands out as a primary global concern, estimated to contribute to millions of premature fatalities and a significant decline in life expectancy. The health impact of air pollution is now seen as comparable to other important global risks, such as unhealthy eating habits and tobacco use (WHO 2021; Hao et al. 2026) Numerous studies have focused on measuring and predicting atmospheric pollutant levels, aiming to better understand their spatiotemporal dynamics and the factors influencing their variability (Shen et al. 2020; Buchholz et al. 2021; Bera et al. 2024; Javadi et al. 2025; Gunwon et al. 2025; Liu and Su 2025; Hao et al. 2026; Muneri et al. 2026). Research on air pollution has expanded steadily, reflecting a global need to better understand pollutant sources, underlying mechanisms, and their impacts.

Atmospheric pollutants such as carbon monoxide (CO) and nitrogen dioxide (NO₂) are key indicators of combustion‐related emissions and significantly affect air quality and public health. CO, a standard tracer of environmental pollution, originates from incomplete fossil‐fuel combustion, the atmospheric oxidation of hydrocarbons, and biomass burning (Buchholz et al. 2021). Studies identify CO as a significant pollutant, evaluated directly through remote sensing analyses alongside future projections (Muneri et al. 2026), in large-scale modeling frameworks and mortality-related (Hao et al. 2026), in monitoring networks linking concentrations to land use and topography (Gunwon et al. 2025), and in urban-scale analyses highlighting thresholds and nonlinearities associated with urban expansion and transport policies (Liu and Su 2025); additionally, CO is used as an input variable for predicting NO₂ (Javadi et al. 2025).

Nitrogen oxides (NOₓ) are produced when atmospheric nitrogen undergoes reactions during high-temperature combustion processes. Among these oxides, nitrogen dioxide (NO₂) is notably significant, as it is predominantly released from the combustion of fossil fuels in various industrial applications. These emissions occur in contexts such as coal- and gas-fired power plants, biomass combustion, electricity generation, and vehicle exhaust systems. (Jion et al. 2023; Rushton and Tate 2025). Vehicular traffic consistently emerges as a key source of NO₂ concentrations, with levels rising with traffic volume (Middya and Roy 2022).

Understanding the sources and impacts of air pollutants is crucial for developing effective strategies to mitigate air pollution and its associated environmental effects. Notably, their patterns and associated health impacts exhibit marked spatial heterogeneity, reflecting the geographic clustering of economic activities and transportation corridors (Dasgupta et al. 2021; Kovács and Haidu 2022).

Because continuous pollutant monitoring remains scarce in many regions, land‐use and land‐cover (LULC) patterns, together with the configuration of the road network, have increasingly been adopted as practical proxies for spatiotemporal variability in atmospheric pollutants (Karroum et al. 2020). Metrics such as impervious‐surface fraction, vegetative cover percentage, and forest extent have proven reliable surrogates for localized emission processes (Hennig et al. 2016; Barbosa and Buriti 2025), while proximity to major roads often correlates with roadside pollutant hotspots (Kovács and Haidu 2022; Metran et al. 2023).

Especially in fire-prone regions during dry seasons, biomass burning is a significant source of atmospheric pollutants. Wildfire events have been shown to substantially increase concentrations of CO and NO₂, contributing to severe air quality degradation (Shrestha et al. 2025). In the Brazilian Amazon–Cerrado transition zone, substantial environmental variability complicates the detection of underlying pollutant trends. Consequently, long‐term monitoring records are essential to distinguish genuine shifts from short‐term fluctuations (Buchholz et al. 2021; Barbosa and Buriti 2025).

Air pollution monitoring can rely on in situ measurements that, when combined with meteorological data, enable robust monitoring and forecasting models (Karroum et al. 2020; Dasgupta et al. 2021; Middya and Roy 2022; Correia Filho et al. 2024). Nonetheless, within expansive regions where ground stations are scarce or nonexistent, satellite observations serve as an effective supplementary resource (Dasgupta et al. 2021; Gu and Zhang 2025; Muneri et al. 2026).

Among remote sensing platforms, the Sentinel-5P mission with the TROPOMI instrument has become a widely used source of trace gas data at both regional and global levels. Measuring tropospheric column density, it offers an effective and consistent way to observe large-scale trends and regional patterns (Bodah et al. 2022; Abdullah et al. 2024; Muneri et al. 2026). Although this approach does not replace sophisticated methods for near-surface estimates, it enables regional monitoring aligned with policy and environmental response scales (Requia et al. 2026).

Moreover, effectively identifying air pollution patterns requires analytical methods capable of decomposing time series into seasonal and long‐term components, particularly when pollutant records exhibit complex patterns (Garbagna et al. 2025). Accordingly, recent advances increasingly favor higher-complexity, data-driven frameworks that explain spatiotemporal variability by integrating satellite observations, such as Sentinel-5P/TROPOMI, with machine-learning algorithms (Javadi et al. 2025; Muneri et al. 2026) and by incorporating urban-form descriptors derived from land-use configuration (Wu et al. 2025).

In this context, employing advanced statistical models, such as the Prophet model (Taylor and Letham 2018), represents an effective strategy for modeling nonlinear trends and intricate seasonal patterns. Such models can reveal insights that traditional methodologies may overlook, thereby enhancing the precision of trend analysis within datasets characterized by significant variability.

Numerous studies have utilized Prophet for both short-term and long-term to analyze trends and forecast air pollution determinants such as SO_2_, NO_2_, O_3_, and CO (Shen et al. 2020; Nath et al. 2021; Bera et al. 2024; Rushton and Tate 2025). The results indicate that it is a reliable method for forecasting future air quality trends, incorporating rigorous statistical models into environmental health research to guide policy and practice (Bera et al. 2024).

Despite advances in air‐pollution monitoring and forecasting, important geographic gaps remain, particularly in large tropical transition zones where rapid land-use change and sparse ground monitoring hinder systematic assessment (Jion et al. 2023; Moura et al. 2024; Moore et al. 2024). Most Brazilian research on atmospheric has historically been conducted at local scales, with a focus on the states of São Paulo and Rio de Janeiro (Maia et al. 2022; Moore et al. 2024). As in most developing countries, ground-based monitoring networks are often sparse, costly, and challenging to maintain, and thus tend to be deployed primarily in heavily industrialized areas or major urban centers where pollution levels are highest (Dasgupta et al. 2021; Moore et al. 2024).

This infrastructural shortfall is especially pronounced within extensive ecological transition zones. The resulting scarcity of ground observations yields incomplete and spatially biased samples, thereby limiting the use of more sophisticated spatial models and impeding a thorough understanding of pollution associated with biomass burning and traffic emissions (Dasgupta et al. 2021; Moura et al. 2024).

Although existing studies indicate that both land-use configuration (particularly deforestation and urbanization) and proximity to highways (as proxies for traffic emissions) are critical determinants of the spatial distribution and intensity of atmospheric pollutants (Paralovo et al. 2019; Marques et al. 2020; Barbosa et al. 2024; Mataveli et al. 2025), it remains unclear how LULC stratification and highway proximity shape CO and NO₂ dynamics or how their integration can improve our understanding of a tropical transition zone complex landscape and its future air‐quality trajectory.

This study addresses the following questions: (i) How do land-use/land-cover patterns and proximity to major roads shape CO and NO₂ distributions across a tropical transition zone? and (ii) To what extent can satellite-based forecasting enhance the capacity to anticipate pollutant trends under ongoing anthropogenic pressures?

To investigate these questions, the study: (i) quantify and compare monthly median CO and NO₂ trends (December 2018–August 2023) across LULC classes and within a 10 km highway buffer; (ii) decompose each subset’s time series into trend and seasonal components with the Prophet model; (iii) forecast pollutant concentrations for September 2024–August 2025, calculate annualized growth rates, and pinpoint areas at greatest risk of air‐quality deterioration.

### Study area

**Fig. 1:**
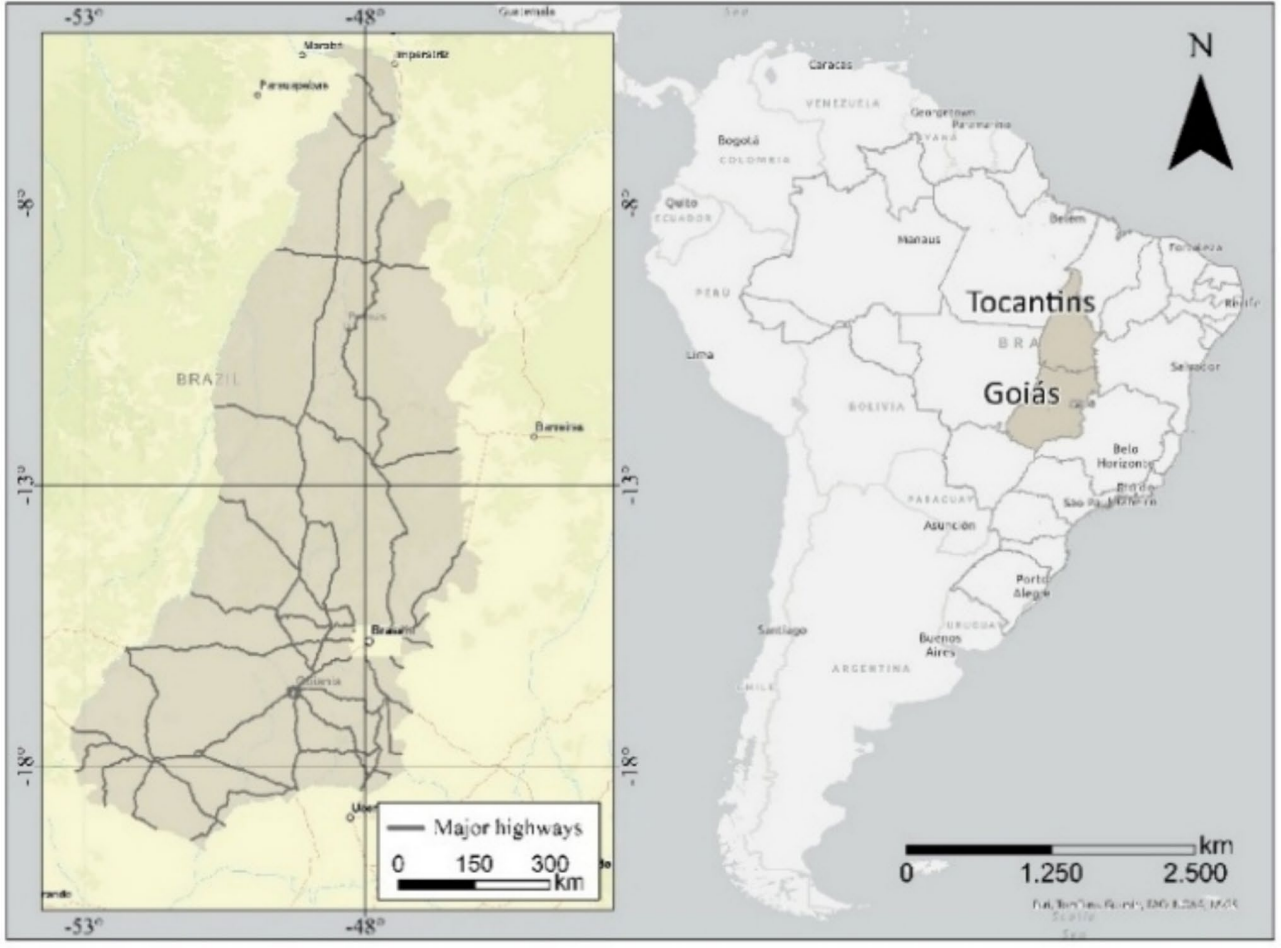
Location of the Brazilian states of Tocantins and Goiás

The study area is located within the South-Central and Amazon Regional Complex and lies in the transition zone between the Amazon and Cerrado biomes, encompassing the Brazilian states of Tocantins and Goiás (Fig. 1). Moreover, it hosts the country’s largest contracted road infrastructure project, involving the duplication of 622 km of highways, with potentially significant environmental implications. The literature consistently indicates that transport infrastructure is a key driver of regional air-quality patterns (Kovács and Haidu 2022), while large-scale road development in ecologically sensitive transition zones can exacerbate land-use change, environmental degradation, and habitat fragmentation, with broader implications for ecosystem resilience and atmospheric pollution (Carvalho et al. 2025).

This region is particularly important due to its rich biodiversity and vital role in carbon and hydrological cycles (Pfaff et al. 2018). Situated within the so-called “Arc of Deforestation,” the region is characterized by pronounced environmental vulnerability and intense anthropogenic pressure (Moura et al. 2024; Correia Filho et al. 2024; Mataveli et al. 2025; Requia et al. 2026). This transition zone has undergone rapid land-use change driven by the expansion of agricultural and livestock frontiers, leading to frequent biomass-burning episodes that release substantial amounts of atmospheric pollutants (Pope et al. 2020).

Studies conducted in the transition zone between the Amazon and Cerrado biomes address a broad spectrum of atmospheric pollutants. PM₂.₅ is widely monitored due to its deep respiratory penetration and strong association with biomass burning and vehicular emissions, while PM₁₀ is commonly assessed, especially in urban and industrial areas, for analyses of its chemical and morphological composition (Moura et al. 2024; Mataveli et al. 2025). In broader assessments, monoaromatic hydrocarbons BTEX (benzene, toluene, ethylbenzene, and xylenes) are found to increase markedly during the dry season, largely driven by biomass pyrolysis across the region (Paralovo et al. 2019).

Specifically, studies integrate remote-sensing technologies, reanalysis products, and localized measurements to assess carbon monoxide (CO) and nitrogen dioxide (NO₂) levels in areas within the Amazon–Cerrado transition zone (Barbosa et al. 2025; Barbosa and Buriti 2025). Abrupt changes in the emission factors of these gases have been identified between the early and mid-dry season (July to September), along with a positive correlation with burned biomass (Barbosa et al. 2025).

Overall, the Amazon–Cerrado transition concentrates key, co-occurring drivers of air-quality variability, yet available evidence remains fragmented and constrained by sparse ground monitoring. By focusing on the South-Central and Amazon Regional Complex (Tocantins and Goiás), this study examines where these pressures converge, enabling assessment of how LULC stratification and highway proximity shape CO and NO₂ patterns with direct relevance for regional environmental and infrastructure planning.

## Method

**Fig. 2:**
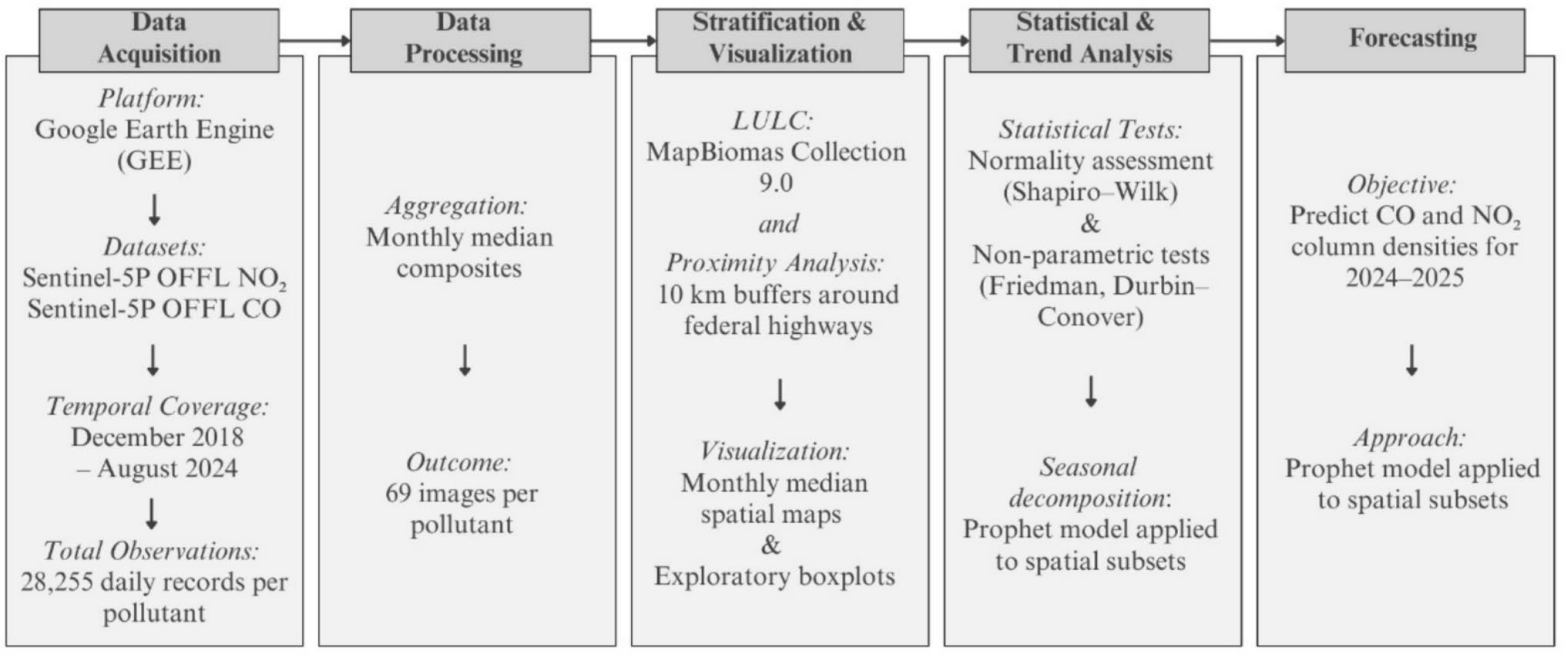
Methodological workflow for CO and NO₂ trend and forecast analysis

The following section details the methodological procedures employed to evaluate the spatial and temporal dynamics of carbon monoxide (CO) and nitrogen dioxide (NO₂) concentrations in relation to land use patterns and proximity to federal highways in the South-Central and Amazon Regional Complex. Figure 2 provides a comprehensive overview of the workflow adopted in the study, with each step elaborated upon in the subsequent subsections.

Data acquisition was performed using the Google Earth Engine platform (Gorelick et al. 2017). All analyses were conducted in Python, using the pandas, SciPy, Matplotlib, and Prophet libraries. Spatial processing was carried out in ArcGIS Pro 3.3.0.

### Data acquisition and processing

The analysis was based on satellite-derived data from the TROPOspheric Monitoring Instrument (TROPOMI) onboard the Sentinel-5P platform, specifically the Level 3 offline (OFFL) products for CO and NO₂ integrated column densities, with a spatial resolution of 1113.2 m. The Level 3 datasets already incorporate quality filtering: for NO₂, the tropospheric column number density band retains only pixels with a quality value greater than 75%, whereas for CO, the typical threshold is 50%. The dataset covers the period from December 1, 2018, to August 31, 2024, totaling 28,255 daily observations for each pollutant.

It is important to note that auxiliary retrieval information, such as averaging kernels, is not provided in the Level 3 products used in this study, which limits a direct linkage between atmospheric column estimates and near-surface concentrations. Since the column–surface relationship is beyond the scope of this study, the use of Level 3 products is methodologically appropriate for our objectives, as it supports regional mapping, characterization of macro-scale patterns, and assessment of monthly or annual trends.

A median filter was applied to the daily dataset to represent the monthly central tendency while minimizing the influence of outliers. This process resulted in a 69-month time series, ensuring a more robust representation of long-term CO and NO_2_ column density trends. This aggregation reduces the influence of short-term meteorological fluctuations, outliers, and irregular sampling associated with cloud screening, producing more stable time series for subsequent statistical testing and forecasting. Consequently, the derived NO₂ and CO metrics are interpreted as predominantly a regional, column-averaged tropospheric signal captured by Sentinel-5P, rather than direct near-surface concentrations or highly localized roadside hotspots.

Data on land use and land cover (LULC) sourced from the MapBiomas platform–Collection 9.0 (MapBiomas 2025a) were employed in this investigation. MapBiomas is a collaborative initiative in Brazil that systematically maps LULC across the entire national territory, achieving a spatial resolution of 30 m.

A compatibilization process was applied to address the differences in spatial resolution between the LULC and pollutants datasets. For each Sentinel pixel, the dominant LULC class was assigned based on the MapBiomas dataset, ensuring consistency in the spatial analysis.

Fire-scar data were obtained from MapBiomas Fire Brazil (Collection 3) (MapBiomas 2025a), which provides annual fire-scar area by biome, state, and municipality across Brazil for each year from 1985 to 2023. This information serves as an indicator of specific instances of elevated atmospheric pollutant concentrations linked to biomass burning.

### Spatial stratification and visualization

**Fig. 3:**
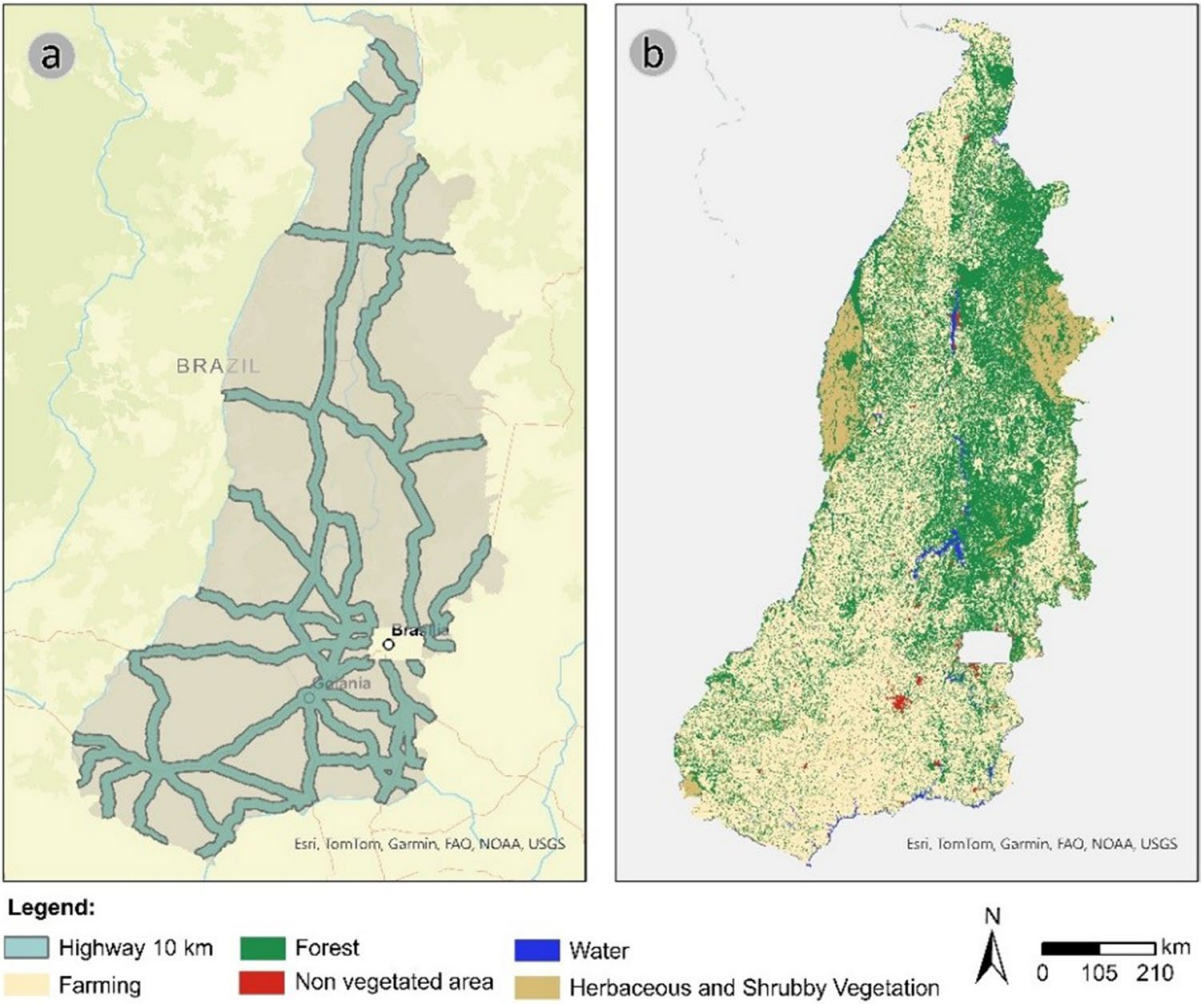
Spatial stratification. **a** Proximity analysis using 10-km buffers around major highways. **b** Land use/land cover (LULC) subsets

Two levels of spatial stratification were adopted to evaluate spatial heterogeneity. Considering the road network’s role as a potential emitter of carbon monoxide, a preliminary analysis was undertaken utilizing a 10-km buffer zone surrounding the federal highways in the study area (Fig. 3a). Furthermore, different land use subsets were analyzed to evaluate their influence: Forest, Farming, Herbaceous and Shrubby Vegetation, Non-Vegetated area, and Water (Fig. 3b). These categories correspond to the standard LULC classes defined by MapBiomas, a Brazilian collaborative land-cover mapping initiative that produces annual, satellite-based land-use/land-cover datasets for Brazil through expert-driven classification and validation (MapBiomas 2025a).

Although these categories are treated as subsets for statistical analysis, it is important to emphasize that they are derived from conceptually distinct classification criteria: the first level of classification is based on infrastructure proximity, in order to capture the direct influence of road traffic emissions; the second level is based on land use and land cover (LULC), to represent different landscape contexts.

Pollutant concentration median values were calculated over the entire study area and separately for each spatial subset, allowing a summary of CO and NO₂ distributions across distinct land use contexts and proximity zones.

Monthly median maps were generated for the study area to evaluate spatial patterns. These maps facilitate the identification of seasonal and spatial variations and potential pollution hotspots. Exploratory boxplots were generated for the whole study area and the 10-km highway buffer to visualize initial spatiotemporal distributions. These visual tools served an exploratory purpose, allowing the identification of distributional patterns and potential anomalies.

### Statistical analysis and temporal trend analysis

Monthly median concentrations of CO and NO₂ for each spatial subset were first subjected to the Shapiro–Wilk test (Shapiro and Wilk 1965) to assess the assumption of normality and determine the appropriateness of non-parametric statistical tests for further analysis. A Friedman test (Friedman 1937) was conducted to compare the pollutant levels across various subsets. This non-parametric test evaluates whether statistically significant differences exist within these distinct spatial contexts. To ascertain which specific pairs of subsets exhibited differences, post-hoc multiple comparisons were performed utilizing the Durbin–Conover method (Conover 1999), with *p*-values adjusted for multiple testing.

Prophet model (Taylor and Letham 2018) was applied to the monthly median CO and NO₂ column density series for each spatial subset. The Prophet model was chosen for its proficiency in handling non-linear trends and irregular seasonal patterns. It offers a robust framework for estimating and comparing trend and seasonal amplitudes, all while avoiding the limitations associated with strict stationarity assumptions (Bera et al. 2024; Taylor and Letham 2018).

### Forecasting

The Prophet model was applied to each spatial subset to project pollutant dynamics from September 2024 to August 2025, generating forecasts of CO and NO₂ column densities based on historical monthly medians. Prophet’s flexibility in handling non-stationary trends, automatic changepoint detection, and customizable seasonal components makes it particularly well-suited for robust forecasting in heterogeneous environments. Moreover, it has been widely adopted in air-quality research for both short- and long-term predictions of key pollutants, demonstrating its versatility and reliability in environmental time-series analysis (Shen et al. 2020; Nath et al. 2021; Bera et al. 2024; Rushton and Tate 2025).

To evaluate the forecast performance, the mean absolute error (MAE), root mean square error (RMSE), and coefficient of determination (*R*^2^) were calculated, providing a comprehensive assessment of the model’s accuracy and predictive capability.

## Results

### Carbon monoxide

**Fig. 4:**
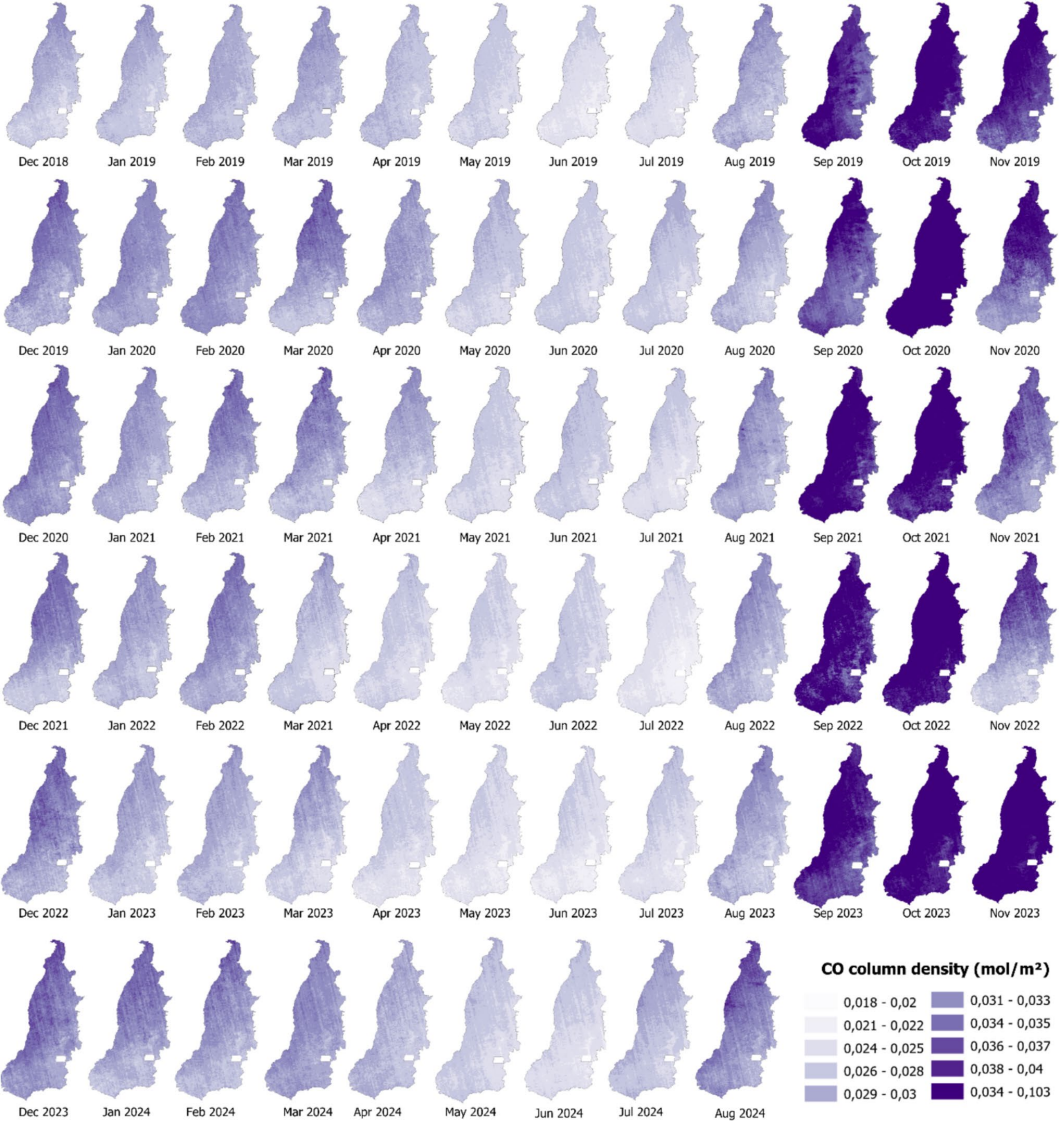
Temporal data and spatial distribution of CO column density

The analysis of carbon monoxide (CO) column density data reveals a seasonal pattern, characterized by annual peaks between September and November. Although within-month spatial variability is evident, CO shows no persistent hotspots or stable spatial gradients; instead, the dominant feature is a seasonal increase during the dry season across the entire region. The mean and minimum CO values have a more stable seasonal pattern, suggesting no extreme variations. Maximum CO values indicate isolated events of high concentration (Fig. 4).

**Fig. 5:**
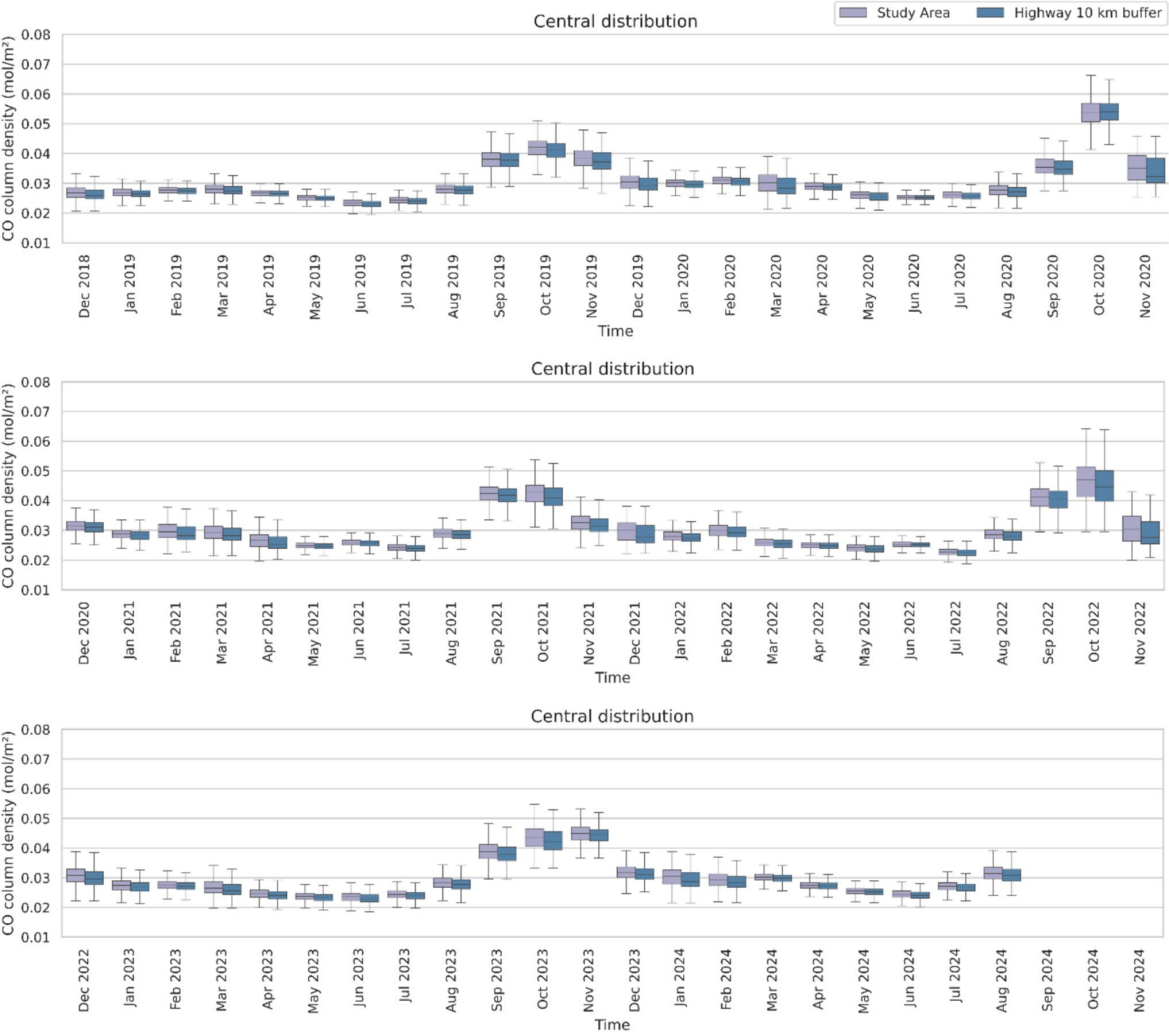
Monthly distribution of carbon monoxide (CO) column density for the study area and highway buffer (10 km)

Figure 5 presents the monthly distribution of carbon monoxide (CO) column density, focusing on areas adjacent to highways. The analysis reveals that CO concentrations in these areas are slightly lower than those observed for the study area, suggesting potential differences in emission sources or dispersion dynamics.

The Shapiro–Wilk test results reject the null hypothesis of normality for the study area and all subsets (*p* < 0.001). This indicates that the data does not follow a normal distribution, suggesting the need for non-parametric statistical approaches for further analyses. The results of the Friedman test (*χ*^2^ = 298; *p*-value < 0.001) confirm the statistically significant differences in the median CO column densities across the different subsets. The Durbin–Conover post-hoc analysis revealed statistically significant differences (*p* < 0.001) for all pairwise comparisons, with two exceptions: the comparison between the highway 10-km buffer and the non-vegetated area (*p* = 0.376) and the comparison between the herbaceous and shrubby vegetation and water subsets (*p* = 0.658) (Table 1).

**Table 1:** Durbin–Conover post-hoc pairwise comparisons for monthly median CO and NO₂ column densities

Comparison	CO statistic	CO *p*-value	NO₂ statistic	NO₂ *p*-value
Study area vs. highway 10-km buffer	13.296	< 0.001	7.044	< 0.001
Study area vs. forest	4.063	< 0.001	3.806	< 0.001
Study area vs. farming	3.693	< 0.001	4.317	< 0.001
Study area vs. herbaceous and shrubby vegetation	8.347	< 0.001	0.966	0.335
Study area vs. non-vegetated area	14.182	< 0.001	14.713	< 0.001
Study area vs. water	7.904	< 0.001	4.942	< 0.001
Highway 10-km buffer vs. forest	17.359	< 0.001	10.850	< 0.001
Highway 10-km buffer vs. farming	9.603	< 0.001	2.727	0.007
Highway 10-km buffer vs. herbaceous and shrubby Veg	21.643	< 0.001	8.010	< 0.001
Highway 10-km buffer vs. non-vegetated area	0.886	0.376	7.669	< 0.001
Highway 10-km buffer vs. water	21.200	< 0.001	2.102	0.036
Forest vs. farming	7.756	< 0.001	8.123	< 0.001
Forest vs. herbaceous and shrubby vegetation	4.284	< 0.001	2.840	0.005
Forest vs. non-vegetated area	18.245	< 0.001	18.518	< 0.001
Forest vs. water	3.841	< 0.001	8.748	< 0.001
Farming vs. herbaceous and shrubby vegetation	12.040	< 0.001	5.283	< 0.001
Farming vs. non-vegetated area	10.489	< 0.001	10.395	< 0.001
Farming vs. water	11.597	< 0.001	0.625	0.532
Herbaceous and shrubby vegetation vs. non-vegetated	22.529	< 0.001	15.678	< 0.001
Herbaceous and shrubby vegetation vs. water	0.443	0.658	5.908	< 0.001
Non-vegetated area vs. water	22.086	< 0.001	9.770	< 0.001

**Fig. 6:**
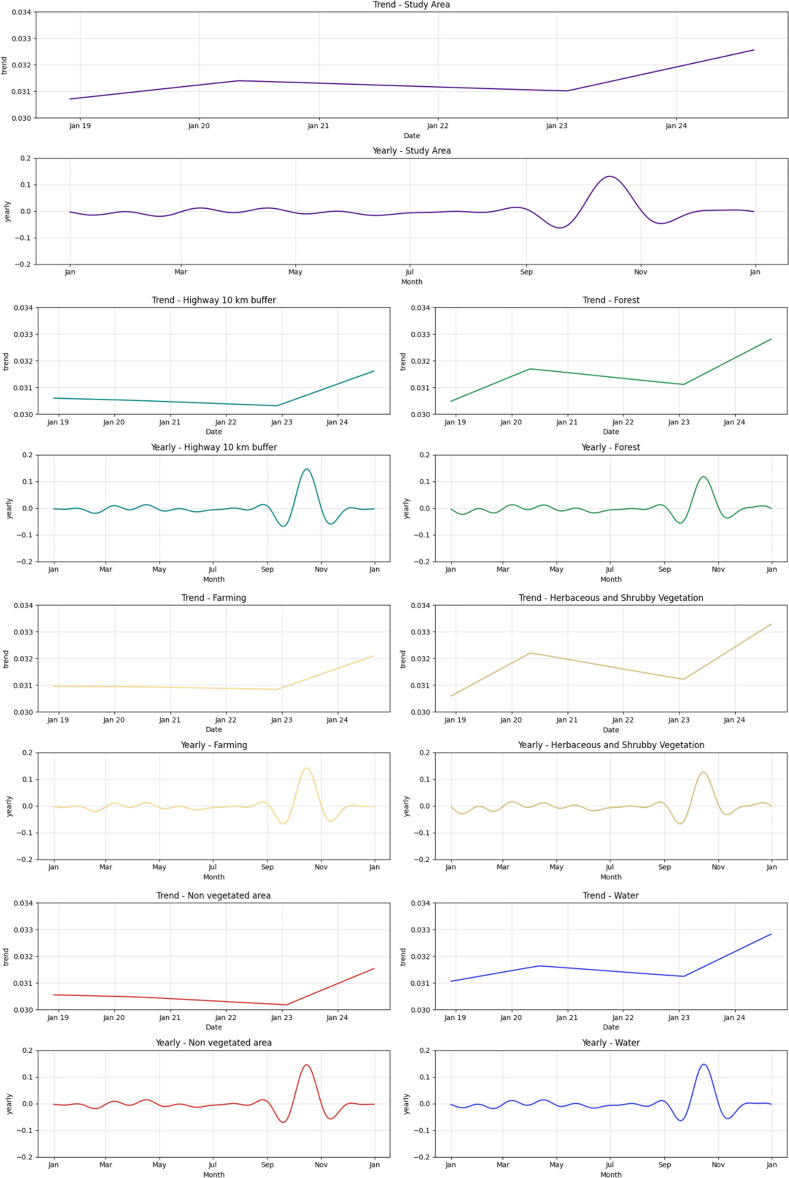
Decomposition of median CO column density time series using the Prophet model (mol/m.^2^)

The decomposition of the median CO column density time series utilizing the Prophet model for the study area, highway proximity, and LULC subsets is illustrated in Fig. 6. The upper panel delineates the long-term trend of the median CO column density for each spatial subset. The lower panel (yearly) depicts the annual seasonal component, highlighting recurrent peak patterns over the study period. Within the model framework, the trend component represents the central trajectory of the time series, whereas the seasonal component captures the periodic fluctuations around this trend over the course of a year. The decomposition analysis offers valuable insights into the long-term variations and recurring seasonal patterns of CO concentrations, along with the distinct patterns of each subset.

The evaluation metrics for the median CO column density Prophet forecasting models, applied to the study area and its subsets, show subtle variations in the statistical performance indicators (MAE, RMSE, and R^2^) across spatial subsets, including land‐use classes and highway proximity zones. However, all results confirm the models' strong forecasting capabilities for the proposed application (MAE < 1.4 × 10⁻^3^ mol/m^2^; RMSE < 1.9 × 10⁻^3^ mol/m^2^; *R*^2^ > 0.9) (Table 2).

**Table 2:** Prophet model performance statistics for predicting the monthly median CO column density for each subset

Subset	MAE (mol/m^2^)	RMSE (mol/m^2^)	*R*^2^
Study area	1.3 × 10^−3^	1.8 × 10^−3^	0.92
Highway 10-km buffer	1.4 × 10^−3^	1.9 × 10^−3^	0.90
Forest	1.2 × 10^−3^	1.7 × 10^−3^	0.93
Farming	1.4 × 10^−3^	1.9 × 10^−3^	0.91
Herbaceous and shrubby vegetation	1.2 × 10^−3^	1.6 × 10^−3^	0.94
Non-vegetated area	1.4 × 10^−3^	1.9 × 10^−3^	0.90
Water	1.3 × 10^−3^	1.8 × 10^−3^	0.93

**Fig. 7:**
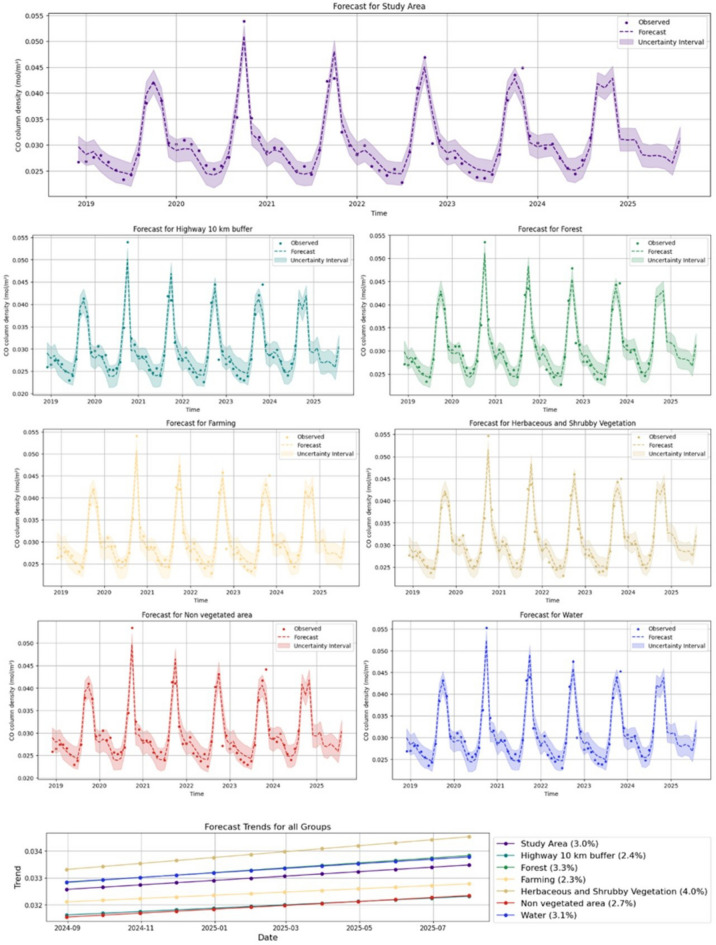
CO column density forecast (2018–2025) e trends (2024–2025)

Figure 7 presents the CO column density forecast generated by the Prophet model for the study area and its various subsets. The figure displays the observed values, predicted values, and the associated uncertainty interval, accounting for potential forecast deviations. Notably, the observed values remain within the uncertainty range, reinforcing the model’s reliability in capturing historical trends. Furthermore, the model effectively identifies a distinct seasonal pattern, highlighting recurring fluctuations in CO concentrations over time.

### Nitrogen dioxide

**Fig. 8:**
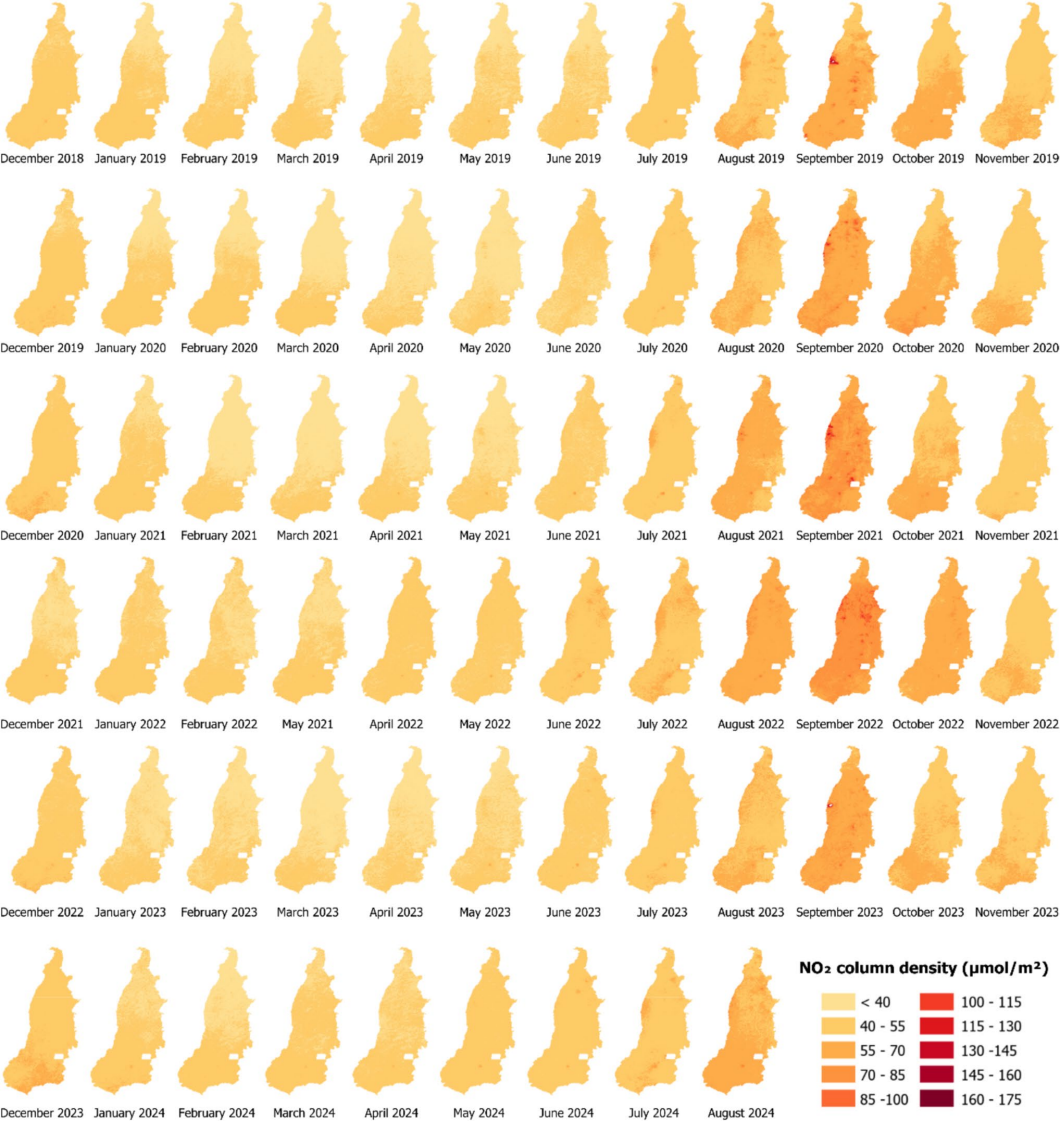
Temporal data and spatial distribution of NO_2_ column density

Figure 8 presents the spatial distribution of NO₂ column densities over the study period. A pronounced seasonal cycle can be identified, with concentrations rising from August through October each year. Moreover, a persistent hotspot is evident over the municipality of Goiânia, which consistently exhibits elevated NO₂ levels in relation to overall values.

**Fig. 9:**
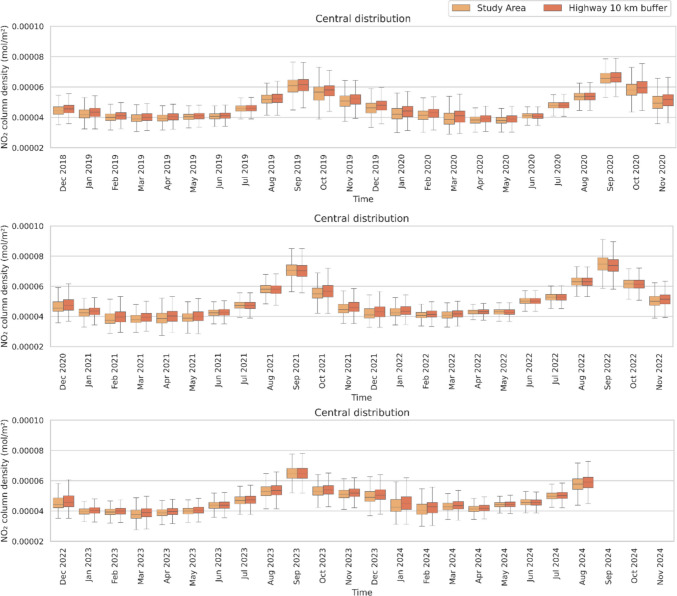
Monthly distribution of nitrogen dioxide (NO_2_) column density for the study area and highway buffer (10 km)

Figure 9 presents the monthly distribution of NO₂ column densities for the whole study area and the highway buffer. Median NO₂ concentrations rise markedly in September each year, reflecting a consistent annual peak. The highway buffer generally exhibits slightly higher medians and a wider interquartile range than the overall study area, indicating that proximity to major roads is associated with elevated NO₂ levels and greater month-to-month variability.

The Shapiro–Wilk test applied to the monthly median for NO₂ rejected the null hypothesis of normality for the overall study area and all spatial subsets (*p* < 0.001), indicating that non-parametric methods are required for subsequent analyses. The Friedman test (*χ*^2^ = 218; *p* < 0.001) demonstrated significant differences in median NO₂ trends among the subsets. Post-hoc pairwise comparisons via the Durbin–Conover procedure showed statistically significant contrasts (*p* < 0.001) for all group pairs except the study area vs. herbaceous and shrubby vegetation (*p* = 0.335), highway 10-km buffer vs. farming (*p* = 0.007), highway 10-km buffer vs. water (*p* = 0.036), forest vs. herbaceous and shrubby vegetation (*p* = 0.005), and farming vs. water (*p* = 0.531) (Table 1).

**Fig. 10:**
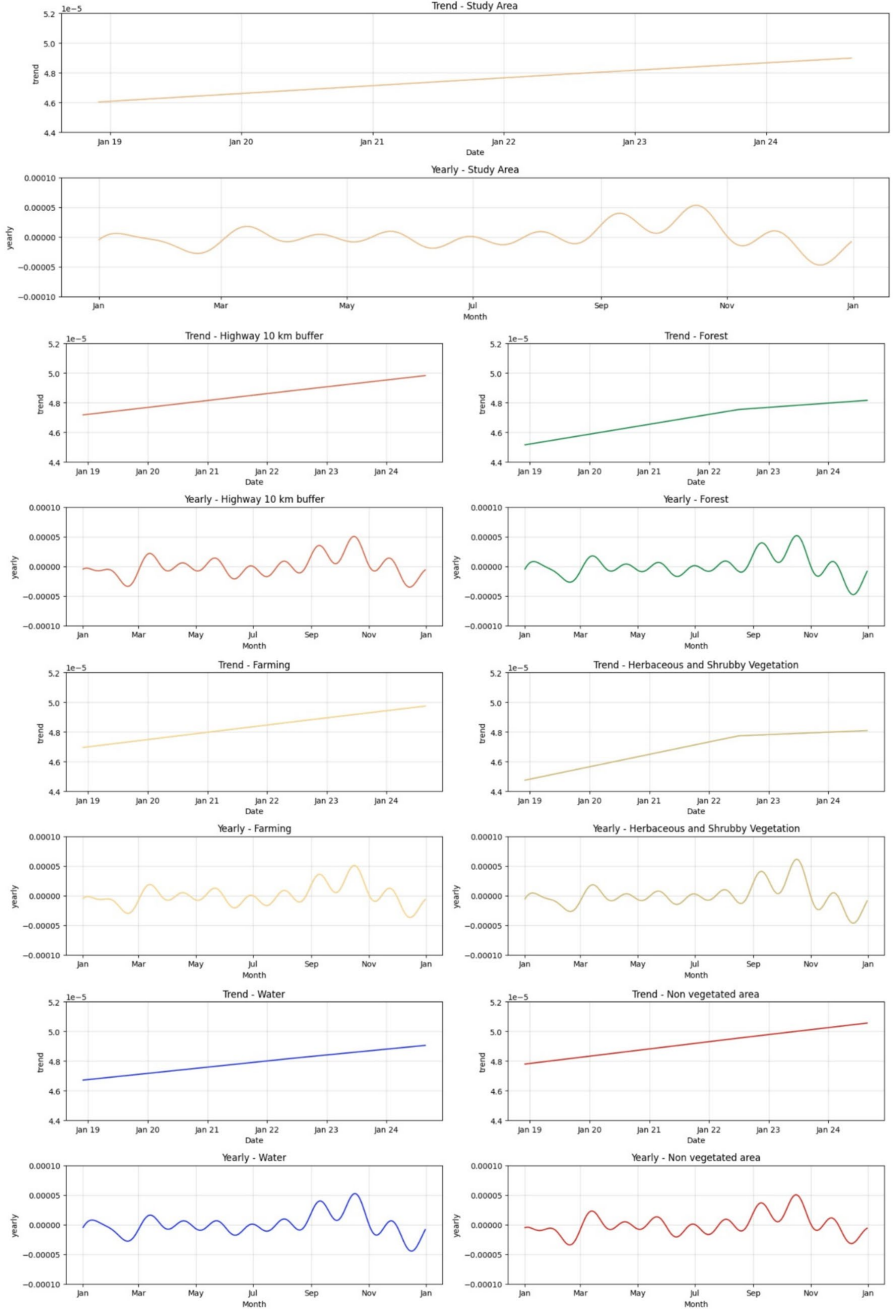
Decomposition of NO_2_ column density time series using the Prophet model (mol/m.^2^)

Figure 10 presents the Prophet decomposition of monthly median NO₂ column densities. All subsets display a positive trend, with NO₂ increasing steadily throughout the period. All subsets share a pronounced annual cycle, peaking between August and October and reaching minima around December–March.

The performance metrics for the Prophet models forecasting monthly median NO₂ column densities across the study area and its spatial subsets underscore their robust predictive accuracy (MAE ≤ 3.0 × 10⁻^6^ mol/m^2^; RMSE ≤ 2.0 × 10⁻^6^ mol/m^2^; *R*^2^ ≥ 0.91) (Table 3).

**Table 3:** Prophet model performance statistics for predicting the monthly median NO2 column density for each subset

Dataset	MAE (mol/m^2^)	RMSE (mol/m^2^)	*R*^2^
Study area	2.0 × 10⁻^6^	2.0 × 10⁻^6^	0.92
Highway 10-km buffer	2.0 × 10⁻^6^	2.0 × 10⁻^6^	0.92
Forest	2.0 × 10⁻^6^	3.0 × 10⁻^6^	0.91
Farming	2.0 × 10⁻^6^	2.0 × 10⁻^6^	0.93
Herbaceous and shrubby vegetation	2.0 × 10⁻^6^	3.0 × 10⁻^6^	0.92
Non-vegetated area	2.0 × 10⁻^6^	2.0 × 10⁻^6^	0.92
Water	2.0 × 10⁻^6^	2.0 × 10⁻^6^	0.92

**Fig. 11:**
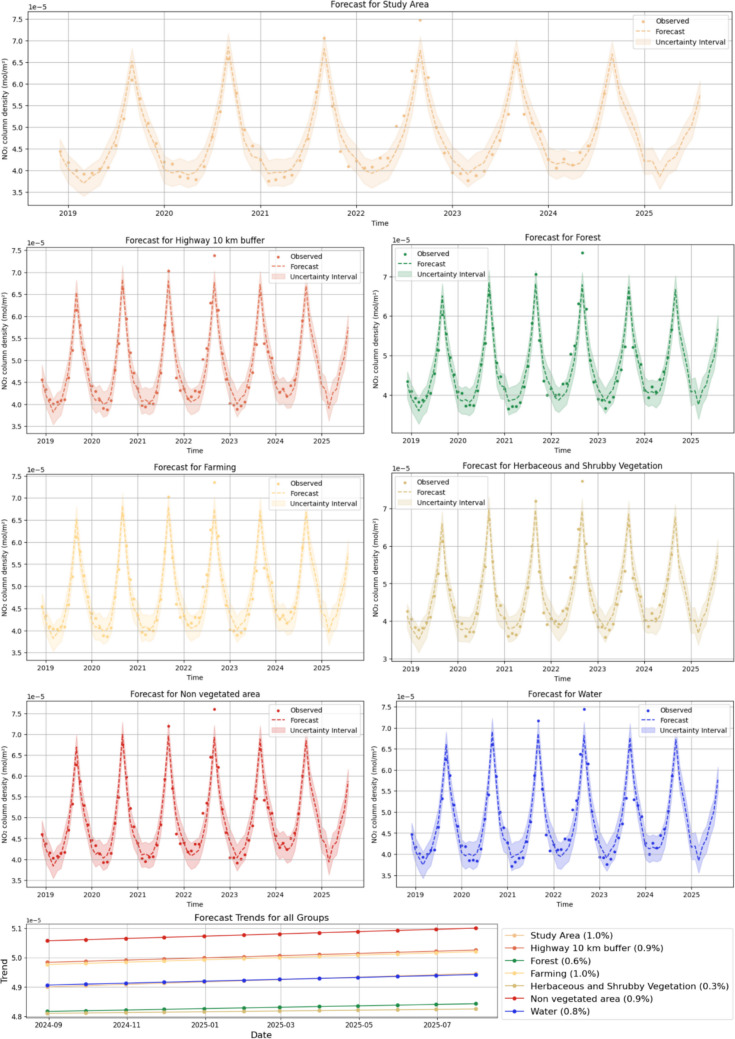
NO_2_ column density forecast (2018–2025) e trends (2024–2025)

Figure 11 exhibits Prophet forecasts of monthly median NO₂ column densities (mol/m^2^) for September 2024–August 2025 and corresponding trend.

## Discussion

### Spatiotemporal characterization of observed CO and NO₂ patterns

The elevated CO and NO₂ concentrations observed between August and October are associated with the peak of the dry season and the regional biomass-burning cycle in the Amazon–Cerrado transition (Silva et al. 2025; INPE 2026). During this period, low soil moisture, reduced relative humidity, and high temperatures promote widespread fires, with NO₂ largely emitted during high-temperature flaming combustion, particularly in savanna vegetation (Pope et al. 2020). In addition, during the wet season, precipitation enhances the removal of atmospheric pollutants, particularly soluble species such as NO₂, through wet deposition and washout processes, contributing to lower observed concentrations outside the dry-season peak (Paralovo et al. 2019).

In parallel, CO is predominantly produced by incomplete and smoldering combustion, which becomes more prevalent as the dry season progresses (Pope et al. 2020). These emission processes are further intensified by stable atmospheric conditions linked to large-scale systems such as the Bolivian High, which suppresses cloud formation and precipitation, limiting vertical mixing and pollutant dispersion (Correia Filho et al. 2024). As a result, both gases accumulate in the regional tropospheric column, explaining their recurrent peaks during the ASO season.

The similarity between CO levels in the highway buffer and the overall study area suggests that biomass burning and meteorological factors were the primary contributors to the increase in CO concentrations during this period. This observation reinforces that these variations are better explained by a land use and land cover (LULC) proxy, which more effectively captures the influence of fire-prone areas. This finding raises the hypothesis that CO emissions may be more significantly influenced by factors beyond those related to anthropogenic activities directly connected to highway operations. Possible contributing elements may encompass biomass combustion, regional atmospheric transport of pollutants, or meteorological conditions that affect the dispersion and accumulation of air pollutants.

In contrast, NO₂ exhibits its highest concentrations in the highway‐proximity and non‐vegetated area subsets—which encompass urban centers—underscoring the dominant role of traffic emissions, localized industrial sources, dense road networks, and urban microclimates. These factors also explain the persistent NO₂ spatial hotspot observed over Goiânia city, a consolidated metropolitan and regional hub, concentrating economic activities, services, and mobility flows. Other municipalities within the study area are comparatively smaller, less urbanized, and do not exhibit the same continuity or magnitude of NO₂ signals in the satellite record. The different spatial patterns of CO and NO₂ trends suggest that while NO₂ dynamics are tightly coupled to urban and roadway emissions, CO levels reflect a broader mix of regional and seasonal influences.

**Fig. 12:**
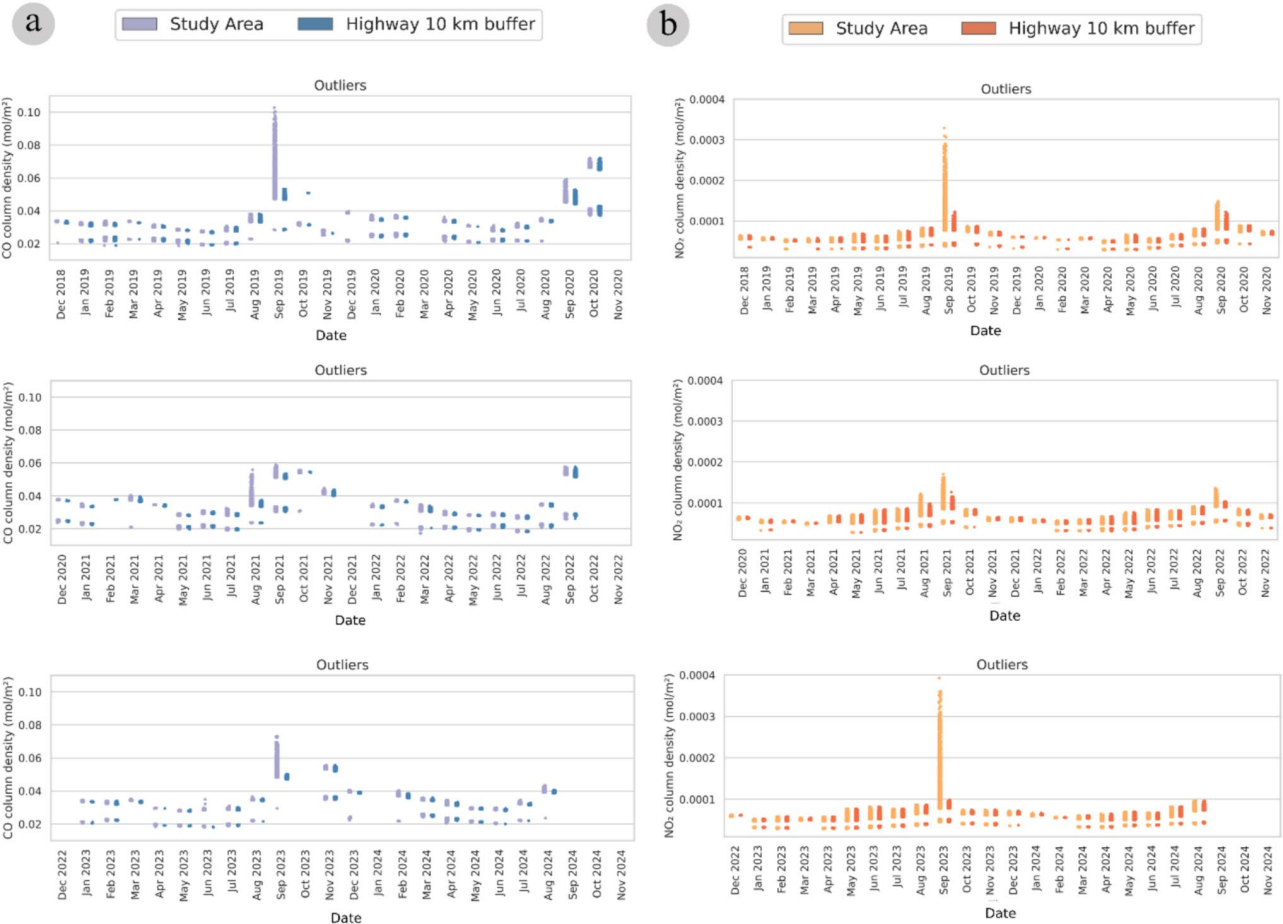
Outlier distributions of monthly (**a**) CO and (**b**) NO₂ column densities for the study area and 10-km highway buffer

Notably, in September 2019, isolated extreme CO and NO₂ concentration events were observed (Fig. 12), reaching the highest values of the entire time series (see also Fig. 5). This pattern aligns with the severe wildfires that occurred in the Amazon during 2019, indicating that reduced environmental monitoring and increased illegal deforestation activities played a crucial role in both fire intensification and elevated CO and NO₂ concentrations (Barlow et al. 2019). The increase in fire activity in 2019 has been attributed to a combination of deforestation, adverse climatic conditions, and shortcomings in the implementation and enforcement of environmental policies (Pivello et al. 2021). Similar dynamics have been observed in other fire-prone regions, where wildfire events led to substantial increases in atmospheric concentrations of CO and NO₂ during the dry season (Shrestha et al. 2025).

In 2020, despite the influence of the COVID-19 lockdown period, during which reduced human activities impacted air quality, CO concentrations exceeded 0.06 mol/m^2^. Except for October 2022 (Fig. 5), this threshold was not surpassed in the subsequent years of the time series, suggesting that the 2020 peak was primarily driven by exceptional environmental conditions rather than direct anthropogenic sources such as transportation and industrial activities, which were significantly reduced during the COVID-19 lockdown. In particular, the peak coincides with an intense fire season, as evidenced by fire-scar records showing one of the highest numbers of fire outbreaks within the analyzed period, predominantly associated with consolidated deforestation areas and native vegetation (INPE 2026). These widespread biomass-burning events provide a plausible explanation for the anomalously elevated CO concentrations observed in that year.

In contrast, NO₂ exhibited more moderate peak magnitudes during 2020. This tempered response underscores the dominant role of continuous traffic and urban emissions in governing NO₂ levels. Consequently, the less pronounced 2020 NO₂ spikes reinforce the conclusion that this pollutant is more tightly coupled to roadway and urban activities. This pattern has also been observed in other regions, reinforcing a strong correlation between transportation infrastructure and NO₂ concentrations during the COVID-19 lockdown (Kovács 2022; Kovács and Haidu 2022).

The concurrent CO and NO₂ peaks observed in September 2023 are consistent with the mid–dry season dynamics of the region (Barbosa et al. 2025; Barbosa and Buriti 2025). Seasonal patterns show recurrent maxima concentration between September and October (Figs. 4 and 8), coinciding with periods of intensified biomass burning, as evidenced by the increase in fire-scar records (Fig. 14) and fire hotspots (INPE 2026), which are known to release large amounts of CO and NO. Additionally, NO₂ exhibits heightened sensitivity to road proximity, with higher medians and stronger growth trends within highway buffers and urban-related land-use classes (Fig. 9), suggesting an additional contribution from increased vehicular emissions associated with post-pandemic economic recovery (Zheng et al. 2025).

### Interpretation of long-term temporal trends

Both CO and NO_2_ level time series exhibit a complex pattern that does not follow a linear monotonic trend and is characterized by fluctuations and abrupt variations over time. The Prophet model’s flexible decomposition of time series into trend and seasonal components makes it particularly well suited to our study, as it allows us to discern distinct trend signatures in monthly median CO and NO₂ column densities across spatial subsets defined by land use/land cover classes and proximity to major highways. Through its automatic changepoint detection and customizable seasonality (Taylor and Letham 2018), Prophet isolates shifts in pollutant trajectories, enabling a direct comparison of how different LULC categories and buffer zones influence air pollutants trends.

Analysis of the CO trend (Fig. 12) for the whole study area reveals two major inflection points: one in early 2021, where the previously rising trajectory levels off and begins a modest decline, and another in early 2023, when this downward phase reverses into renewed growth. Comparable temporal patterns appear in the forest, herbaceous and shrubby vegetation, and water subsets, albeit with varying magnitudes.

**Fig. 13:**
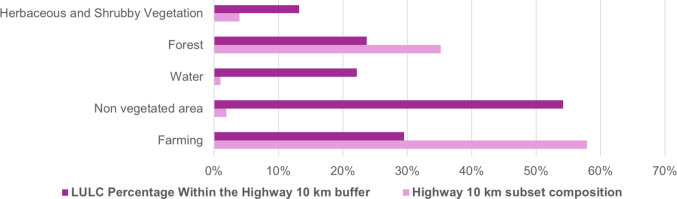
Percentage of each LULC class from the entire study area located within the 10-km highway buffer and the internal LULC composition of the highway-buffer subset itself

The herbaceous and shrubby vegetation subset exhibits the steepest trend fluctuations—likely reflecting the impact of intense wildfire events, particularly during 2019—while the farming and non-vegetated subsets follow a distinct trajectory characterized by more abrupt shifts. The abrupt shifts observed in the farming and non-vegetated subsets reflect their strong sensitivity to rapid changes in anthropogenic activity. These areas are largely concentrated near major highways (Fig. 13), making them highly responsive to post-pandemic increases in traffic intensity and economic activity from 2023 onward.

**Fig. 14:**
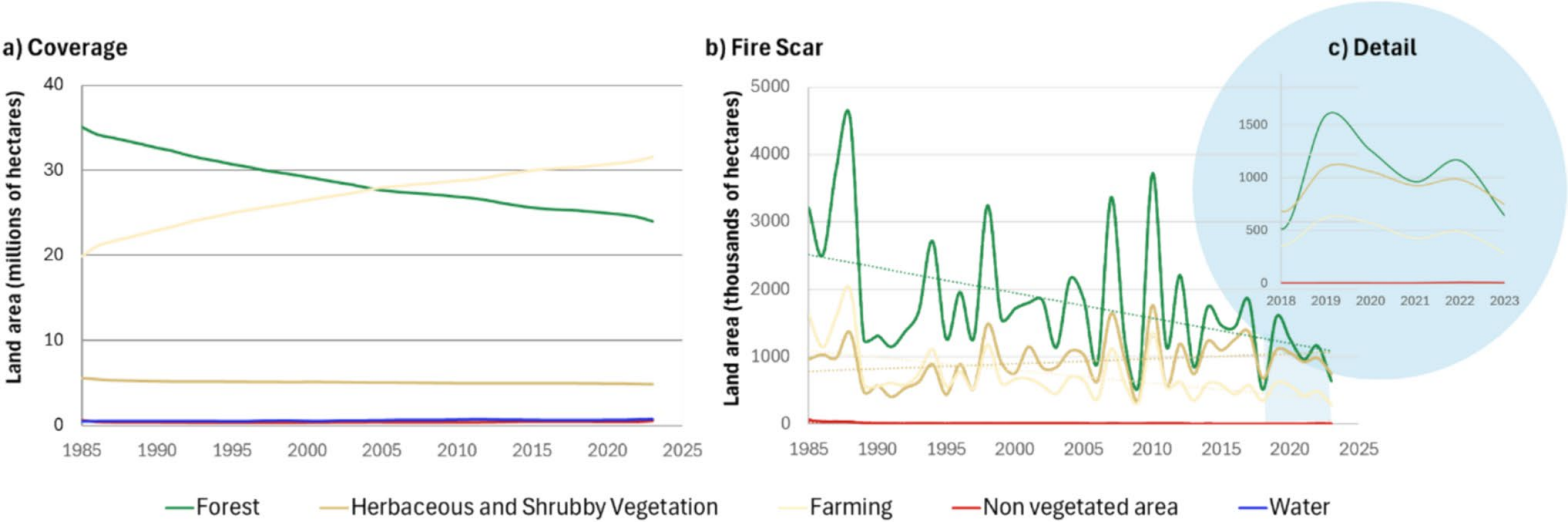
**a** Spatial distribution of LULC classes across the study area. **b** Temporal evolution of fire scars detected within these LULC classes from December 2018 to August 2024

In addition, intensified dry-season fires in agricultural and transitional landscapes contributed to episodic CO enhancements, producing steeper trend inflections compared to the smoother evolution of the study-area average (Fig. 14a).

Three distinct clusters emerge from the NO₂ trend analysis: (i) highway buffer, non-vegetated area, and farming exhibit the steepest and most sustained upward trends; (ii) water show intermediate, steady growth with no notable shift in trend magnitude over the study period; and (iii) forest and herbaceous and shrubby vegetation subsets exhibit the lowest trend slopes, with a pronounced deceleration in growth rates beginning in 2022. These groupings reflect analogous trend dynamics within each cluster and highlight how land cover and proximity to major roadways modulate the temporal behavior of NO₂. Although these categories are treated as subsets for statistical analysis, they are based on conceptually distinct classification criteria. Since 30% of the farming area and 54% of the non‐vegetated area lie within the 10-km highway buffer (Fig. 13), the highway‐proximity subset inherently reflects the trend characteristics of these land‐cover classes. Land‐cover specific processes, such as post‐fire regrowth and surface properties, exert a more substantial control over CO temporal dynamics than mere road adjacency. Conversely, NO₂ concentrations are highly sensitive to highway proximity, underscoring the dominant influence of vehicular emissions and urban infrastructure along these transport corridors on NO₂ temporal behavior.

These observations align with the documented decline in forest cover and the concurrent expansion of herbaceous and shrubby vegetation in the study area, reflecting an ongoing vegetation‐transition process (MapBiomas 2025a, 2025b). Also, the variation in fire scars across different land cover classes reveals a notable increase within forests, particularly in 2019 (Fig. 14). Thus, recent fire events have significantly impacted forest regions, suggesting a deforestation-driven landscape change and the growing influence of fire disturbances in shaping land cover dynamics over the past decades.

### Forecasting CO and NO₂ dynamics (2024–2025)

Although CO levels have declined (Fig. 5), the trends show a recent resurgence (Fig. 6), indicating a return to an upward trajectory expected to intensify further in the forecasted period (Fig. 7). The analysis reveals rising CO concentration trends estimated for 2024–2025 across all subsets. The smallest growth trends were observed in areas near highways and non-vegetated regions, suggesting a lower impact of road traffic and urban emissions on overall CO levels. In contrast, the most pronounced increasing trends were identified in forest areas (3.3%) and herbaceous and shrubby vegetation (4%), reinforcing the strong influence of biomass burning and land cover changes on CO emissions.

Regarding the projected trends for NO₂ levels, the non-vegetated area subset stands out with the highest absolute trajectory throughout the forecast period, reflecting both an elevated baseline column density and a persistent upward drift. The results indicate that both the study area and the specific farming subsets demonstrate the most significant relative growth rates (1.0% y⁻^1^), while the highway buffer and non-vegetated area show slightly lower but still pronounced increases (0.9% y⁻^1^). These patterns underscore the vulnerability of open and agriculturally managed landscapes to escalating NO₂ pollution. In contrast, the Forest (0.6% y⁻^1^) and herbaceous and shrubby vegetation (0.3% y⁻^1^) subsets display the most moderate growth, indicative of the buffering capacity provided by dense canopy cover and active vegetation uptake in mitigating NO₂ accumulation.

Although the use of Prophet in air pollution studies remains limited compared to more established deep learning or autoregressive models, its adoption has shown promising results, especially in urban monitoring contexts (Shen et al. 2020; Middya and Roy 2022). This study precedes the application of Prophet in the South-Central and Amazon Regional Complex, providing initial evidence of the model’s robust performance (*R*^2^ > 0.9) and effectively capturing both seasonal and long-term dynamics. These results support its suitability for environmental time-series applications, especially in regions characterized by high temporal variability and data scarcity.

## Conclusions

### Key findings

The proposed framework, which combines exploratory analysis, inferential statistics, and predictive modeling, offers a comprehensive understanding of spatial and temporal dynamics trends.

The results indicate that land-use/land-cover patterns and road proximity significantly influence the spatial distribution and seasonal behavior of atmospheric CO and NO₂. CO levels were more strongly associated with areas affected by biomass burning, especially in herbaceous and shrubby vegetation zones, while NO₂ concentrations were higher near urbanized areas and highway corridors. These findings reinforce the importance of integrating land systems and infrastructure context into air-quality assessments in tropical transition regions.

To address the second research question, the Prophet forecasting model, using remote sensing data, effectively captured non-monotonic trends, estimated seasonal variations, and predicted pollutant levels one year in advance. Its performance in this context shows notable accuracy. The model’s versatility, which does not depend on meteorological data, is particularly important for analyzing extensive regions with limited data availability, such as the Amazon-Cerrado transition zone. This capability enables robust trend estimation and forecasting, even with scarce data context.

From a policy perspective, the results can support targeted actions to mitigate the increasing trend of atmospheric pollutants. Because CO and NO₂ exhibit distinct spatial dynamics and source signatures, the proposed framework supports pollutant-specific and targeted policy recommendations. CO patterns suggest support priorities in fire management, vegetation conservation (notably in herbaceous/shrubland areas with higher projected growth), and stronger control of illegal deforestation and severe fire events, ideally coordinated at a regional scale. NO₂ patterns motivate targeted transport and infrastructure measures, including hotspot-oriented mobility planning (e.g., Goiânia), vehicle-emission controls along major highways, and evaluation of potential agricultural contributions where growth rates are non-negligible. Finally, the results highlight the value of anticipatory tools: 1-year-ahead forecasting (e.g., Prophet) can support proactive air-quality management even under limited meteorological data availability, while integrating LULC context with transport infrastructure improves the policy relevance of regional air-quality assessments in tropical transition zones.

### Limitations

This analysis is based on monthly median composites of Sentinel-5P column products and on relatively broad spatial units, including land-use/land-cover strata and a 10-km buffer around federal highways. As a result, the derived metrics primarily reflect regional, column-averaged tropospheric concentrations rather than localized near-surface or near-road pollution levels. Although spatial and temporal aggregation reduces short-term variability, cloud-related data gaps, and observational noise, it does not resolve the fundamental distinction between satellite columnar observations and surface-level air quality. This approach was adopted because the study region covers a large spatial extent and lacks dense ground-based monitoring networks and high-resolution meteorological data, making monthly satellite composites a practical and scalable solution for identifying macro-scale trends and regional patterns. Nevertheless, the results should be interpreted as indicators of regional atmospheric behavior and are not intended to replace in situ measurements or coupled chemistry–meteorology models for fine-scale exposure assessment or detailed causal attribution.

### Future work and research opportunities

Future research should incorporate the use of averaging kernels in the processing of TROPOMI data, allowing a more accurate linkage between column-integrated densities and near-surface pollutant concentrations while reducing reliance on a priori vertical profiles. In addition, expanding the analysis to include ground-based air-quality measurements would provide an independent validation of the temporal trends detected by satellite observations. Particularly, future studies should investigate highly urbanized areas, such as Brasília, separately, considering their distinct emission dynamics and the stronger influence of urban activities on atmospheric pollutant concentrations.

Additionally, other pollutants such as sulfur dioxide (SO₂), methane (CH₄), and ozone (O₃) can be incorporated into the analysis to provide a more comprehensive understanding of atmospheric composition and pollution dynamics. A multivariate analysis could also explore the interactions between different pollutants and their potential relationships with land use, meteorological conditions, and anthropogenic activities.

## Data Availability

The Sentinel-5P TROPOMI datasets used in this study—NO₂ (COPERNICUS/S5P/OFFL/L3_NO₂) and CO (COPERNICUS/S5P/OFFL/L3_CO) products—are publicly available through the Copernicus Open Access Hub (https://browser.dataspace.copernicus.eu) and the Google Earth Engine Data Catalog (https://developers.google.com/earth-engine/datasets/catalog/sentinel-5p). Land-use and land-cover data were obtained from MapBiomas Coverage and transitions statistics by biomes–MapBiomas Brasil Collection 9, available at 10.58053/MapBiomas/7VJZWK. Fire scar data were sourced from MapBiomas Fire Brazil–Collection 3, available at 10.58053/MapBiomas/W2KSHU. Additional derived datasets—including forecast outputs, LULC stratifications, and highway proximity zones—are available from the corresponding author upon reasonable request.
